# A Case of an Invisible Pancreatic Neuroendocrine Tumor Decoded by Endoscopic Ultrasound

**DOI:** 10.7759/cureus.109296

**Published:** 2026-05-20

**Authors:** Jenny Joseph, Syed Alishan Nasir, Rakhee Mangla

**Affiliations:** 1 Internal Medicine, Norwalk Hospital, Norwalk, USA; 2 Gastroenterology, Norwalk Hospital, Norwalk, USA

**Keywords:** endoscopic ultrasound (eus), eus-fna pancreatic lesions, negative cross-sectional imaging, pancreatic insulinoma, pancreatic neuroendocrine tumors

## Abstract

Endoscopic ultrasound (EUS) is a highly sensitive modality of imaging for detecting small pancreatic masses that may be missed on other cross-sectional imaging. We report a 61-year-old woman who presented with recurrent symptomatic, predominantly postprandial, hypoglycemic episodes, which were confirmed on continuous glucose monitoring. Pertinent medical history included a remote use of tirzepatide, a dual glucagon-like peptide-1 (GLP-1)/glucose-dependent insulinotropic polypeptide (GIP) receptor agonist, for weight loss. Mixed-meal testing performed four months after discontinuation of tirzepatide demonstrated hypoglycemia with inappropriately normal insulin and C-peptide levels and a high proinsulin level. Contrast-enhanced computed tomography (CT) of the abdomen and pelvis was negative for pancreatic masses. Due to high clinical suspicion for an insulinoma, EUS was pursued, and a 12-mm hypoechoic mass was identified in the pancreatic body. EUS-guided fine needle biopsy demonstrated a well-differentiated, World Health Organization (WHO) grade 1 pancreatic neuroendocrine tumor with immunostaining consistent with insulinoma. Later, she underwent pancreatic tumor enucleation with complete resolution of symptoms postoperatively. This case highlights the diagnostic utility of EUS in patients with biochemically suspected insulinoma and negative cross-sectional imaging. EUS not only improves mass detection but also facilitates tissue acquisition, supporting timely surgical management of small pancreatic neuroendocrine tumors.

## Introduction

Endoscopic ultrasound (EUS) was first introduced for better visualization of the pancreas compared to transabdominal ultrasound by overcoming the obscurities caused by overlying abdominal gas. Since the pancreas is a retroperitoneal organ, pancreatic neoplasms are challenging to diagnose. The major clinical indications for EUS include the detection of common bile duct stones, evaluation of pancreatic exocrine and endocrine neoplasms, and guidance of fine-needle aspiration (FNA) of these lesions [1]. Pancreatic neuroendocrine tumors (PNETs) are classified as functioning and non-functioning, and the most common functioning PNETs are insulinomas with an incidence of almost four cases per million per year [2]. With the widespread use of incretin analogs, such as glucagon-like peptide-1 (GLP-1)/glucose-dependent insulinotropic polypeptide (GIP) receptor agonists for weight loss, episodes of hypoglycemia have become more common in non-diabetic patients as well. The use of GLP-1/GIP receptor agonists in isolation is less likely to cause hypoglycemia, but it can uncover occult insulinomas due to its exaggerated effect on the tumor cells composed of pancreatic beta cells, causing hypoglycemic episodes. Small insulinomas can be challenging to detect on conventional imaging; however, EUS helps identify these masses with accurate anatomic localization through the high spatial resolution it offers. EUS has a sensitivity of around 94% in the detection of biochemically proven insulinomas [3]. We present a case of a middle-aged female with a history of incretin analog usage with biochemical features of insulinoma but negative imaging findings who was found to have a small pancreatic body insulinoma through EUS.

## Case presentation

During one of her regular endocrinology visits, a 61-year-old female with a history of Graves' disease and thyroid nodules reported episodes of shaking and feeling unwell after meals. She also stated that her blood glucose levels ranged between 50 and 60 mg/dL during these episodes, and her symptoms resolved after drinking carbonated drinks. Interestingly, these episodes of documented hypoglycemia occurred primarily after meals and occasionally following physical activity. Notably, she had initiated tirzepatide 2.5 mg for weight loss one month before the onset of hypoglycemic symptoms. After the dose was increased to 5 mg, the episodes became progressively more frequent, leading to gradual tapering and eventual self-discontinuation of the medication after five months of therapy. However, her symptoms persisted for four months after discontinuation, prompting her to report them to her endocrinologist. She also reported a history of lymphoma in her sister. She was hemodynamically stable with normal vitals. Labs were remarkable for fasting glucose 57 mg/dL and normal hemoglobin A1C at 5%. She was started on a continuous glucose monitor (CGM), which registered blood glucose levels, ranging within 50-60 mg/dL, in both fasting and postprandial states. She was then hospitalized to undergo a mixed meal tolerance test with serial measurement of insulin, C-peptide, and proinsulin levels after a mixed-nutrient liquid meal. The mixed meal tolerance test was positive. Despite a symptomatic hypoglycemic state with a blood glucose of 54 mg/dL, the corresponding insulin and C-peptide levels remained unsuppressed at 17.9 uU/mL and 3 ng/mL, respectively, which are inappropriately normal and diagnostic of endogenous hyperinsulinism (Table 1). Additionally, proinsulin level was significantly elevated at 12.9 pmol/L, suggestive of insulinoma, and she did not have antibodies to insulin to suggest type 1 diabetes. She underwent a contrasted CT study of the abdomen and pelvis (CT A/P) due to suspicion for insulinoma which was negative for intra-abdominal masses (Figure 1).

**Table 1:** Laboratory values on initial presentation and the mixed meal tolerance test

Laboratory test	Result	Reference range
Initial Presentation		
Hemoglobin A1c	5%	4-5.6%
Fasting glucose	57 mg/dL	70-99 mg/dL
Mixed Meal Tolerance Test		
Fasting glucose	54 mg/dL	70-99 mg/dL
Insulin	17.9 uU/mL	2.6-24.0 uU/mL
C-peptide	3 ng/mL	1.1-4.4 ng/mL
Proinsulin	12.9 pmol/L	< 7.2 pmol/L
Insulin antibody	< 0.4 U/mL	< 0.5 U/mL

**Figure 1:**
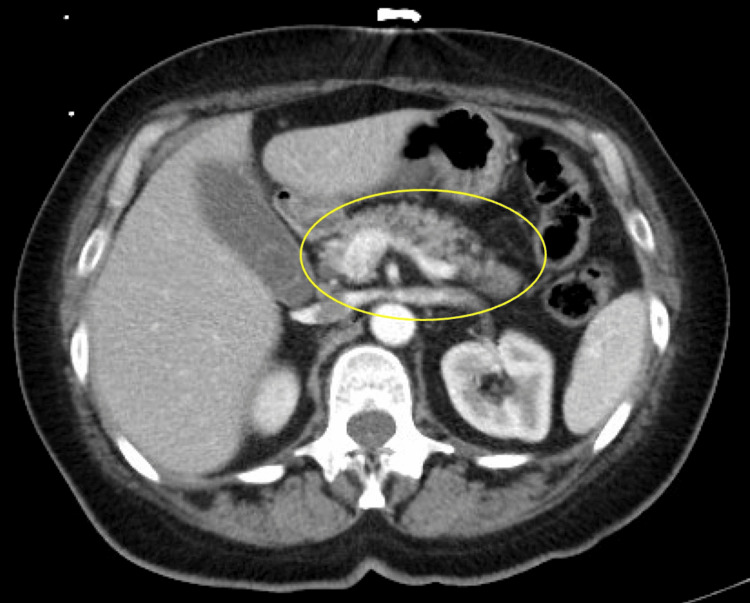
Contrasted CT abdomen/pelvis (late arterial or pancreatic phase) showing the pancreas (yellow circle) with no structural abnormality

Although contrasted CT A/P was negative for masses, she was referred to a gastroenterologist for EUS due to high clinical suspicion for insulinoma. On EUS, a round hypoechoic mass measuring 12.3 mm x 12.8 mm was identified in the pancreatic body with normal appearance of the rest of the pancreatic parenchyma, and the pancreatic duct (Figure 2). An FNA of the mass was performed (Figure 3).

**Figure 2:**
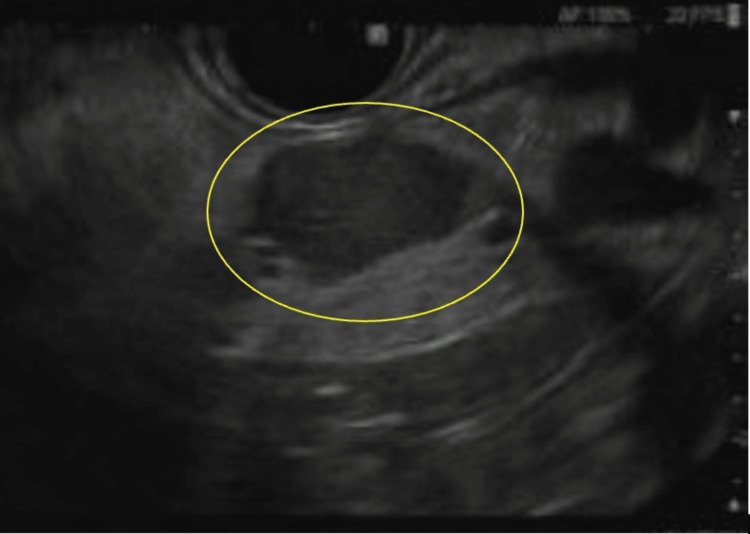
Endoscopic ultrasound image showing the hypoechoic pancreatic mass (yellow circle)

**Figure 3:**
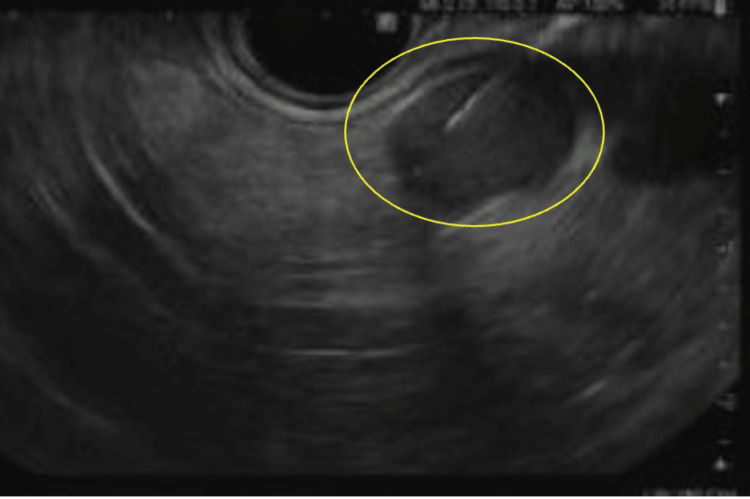
Endoscopic ultrasound image showing the hypoechoic pancreatic mass (yellow circle) with the fine needle aspiration needle (central hyperechoic line)

Pathology of the pancreatic mass showed few scattered tumor cells with immunostaining positive for synaptophysin, suggestive of the WHO grade 1 well-differentiated neuroendocrine tumor (Figures 4A-4C), consistent with an insulinoma. She then underwent pancreatic tumor enucleation with surgical pathology confirming a WHO grade 1 well-differentiated neuroendocrine tumor. During a follow-up visit one month after the procedure, she reported complete resolution of hypoglycemia symptoms with CGM readings of normal fasting and post-prandial blood glucose levels.

**Figure 4:**
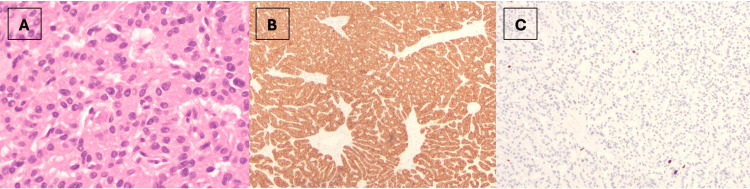
(A) H&E stain showing nests of cells with mild cytologic atypia and low nuclear cytoplasmic ratio, original magnification 400x. (B) Immunohistochemistry showing cells strongly positive for synaptophysin, original magnification 100x. (C) Ki-67 index of less than 1% supports a WHO grade 1 neuroendocrine tumor, original magnification 100x H&E - Hematoxylin and eosin; Ki-67 - It is a cell proliferation marker that is utilized in WHO grading of neuroendocrine tumors. A Ki-67 index of < 3% indicates a WHO grade 1 neuroendocrine tumor.

## Discussion

Insulinomas and gastrinomas are the most common neuroendocrine tumors, but one of the key differentiations is that 99% of insulinomas are in the pancreas, whereas gastrinomas occur in extrapancreatic sites, such as the duodenum or stomach, in 28-44% of cases [4-6]. These tumors are routinely localized on pre-operative non-invasive tests, including ultrasonography and CT, but smaller masses are not identified in 10-30% of cases [4]. EUS can help in detecting pancreatic masses as small as 2-3 mm in diameter [4], such as in our patient. This not only helps in surgical planning but also in obtaining biopsy samples using FNA [7].

In our patient, the association between the onset of symptoms and the initiation of tirzepatide raised concern for possible medication-induced hypoglycemia, although it is an uncommon adverse effect and is usually present with concomitant use of sulfonylureas or insulin. Moreover, the persistence of symptoms for four months after its discontinuation supported an alternative etiology because the half-life of tirzepatide is about five days [8] with complete elimination in three to four weeks. Hypoglycemic episodes with inappropriately normal or high insulin and C-peptide levels and a high proinsulin level are suggestive of insulinoma due to the inability of the tumor cells to process the prohormone, leading to its immature release and high plasma proinsulin levels. Additionally, there are cases in the literature highlighting the unmasking of insulinomas by GLP-1/GIP receptor agonists due to their insulinotropic effects on pancreatic beta cells [9,10].

While imaging modalities, including transabdominal ultrasound, CT, and magnetic resonance imaging (MRI), were estimated to have a sensitivity of 29-60% in the detection of PNETs, EUS was noted to have a sensitivity of 88% and specificity of 98% [11]. The results of a retrospective study by Rosch et al. reported a sensitivity of approximately 80% in patients who had negative transabdominal ultrasound or CT. It also highlighted that this might be an underestimate because of technical failures with the echoendoscope [4]. A prospective study by Schumacher et al. showed that the diagnostic accuracy of EUS in insulinomas was dependent on the location of the tumor [12]. The sensitivity for those in the head of the pancreas was 83%, whereas it was 37% for those in the tail of the pancreas. There was no significant difference in the median diameters and volumes between the detected and undetected tumors. Although EUS is highly sensitive in the detection of insulinomas and intrapancreatic gastrinomas, its sensitivity for duodenal wall gastrinomas is as low as 40% [13]. Hence, the most recent guidelines suggest the use of gallium-68 DOTATATE PET/CT as first-line in the detection of all PNETs, especially extra-pancreatic gastrinomas [14].

EUS is limited by the high operator dependency and the resultant observer variability. It is also a highly skilled procedure, demanding at least a hundred procedural exposures to gain proficiency. Additionally, large hiatal hernias and prior abdominal surgeries can preclude complete visualization of the pancreas. Nonetheless, it is a sensitive diagnostic tool that also allows tissue sampling through EUS-guided FNA with a sensitivity of 87% [15]. This case demonstrates the importance of a stepwise diagnostic approach, wherein EUS should be strongly considered in patients with biochemical evidence of PNET and negative cross-sectional imaging.

## Conclusions

Persistent hypoglycemia in patients using incretin analogs, such as GLP-1/GIP receptor agonists, should be investigated despite the possibility of medication-induced hypoglycemia, as their usage can unmask underlying insulinomas due to their insulinotropic effects on pancreatic beta cells. In cases with high clinical and biochemical suspicion, EUS must be pursued despite negative cross-sectional imaging. In current practice, EUS is the best available technique for the diagnosis of small pancreatic tumors (< 3 mm in diameter) with a sensitivity greater than that of CT, percutaneous ultrasound, or MRI. However, it is important to remember that EUS has reduced sensitivity in the detection of duodenal wall neuroendocrine tumors. EUS-guided FNA is useful in distinguishing inflammatory and malignant masses without the negative effects of tumor seeding.
